# Combination Lenalidomide and Azacitidine: A Novel Salvage Therapy in Patients Who Relapse After Allogeneic Stem-Cell Transplantation for Acute Myeloid Leukemia

**DOI:** 10.1200/JCO.18.00889

**Published:** 2019-01-17

**Authors:** Charles Craddock, Daniel Slade, Carmela De Santo, Rachel Wheat, Paul Ferguson, Andrea Hodgkinson, Kristian Brock, Jamie Cavenagh, Wendy Ingram, Mike Dennis, Ram Malladi, Shamyla Siddique, Francis Mussai, Christina Yap

**Affiliations:** ^1^ Queen Elizabeth Hospital, Birmingham, United Kingdom; ^2^ University of Birmingham, Birmingham, United Kingdom; ^3^ University Hospital North Staffordshire, Stoke-on-Trent, United Kingdom; ^4^ St Bartholomew’s Hospital, London, United Kingdom; ^5^ University College Hospital, Cardiff, United Kingdom; ^6^ The Christie Hospital, Manchester, United Kingdom

## Abstract

**PURPOSE:**

Salvage options for patients who relapse after allogeneic stem-cell transplantation (allo-SCT) for acute myeloid leukemia (AML) and myelodysplasia (MDS) remain limited, and novel treatment strategies are required. Both lenalidomide (LEN) and azacitidine (AZA) possess significant antitumor activity effect in AML. Administration of LEN post-transplantation is associated with excessive rates of graft-versus-host disease (GVHD), but AZA has been shown to ameliorate GVHD in murine transplantation models. We therefore examined the tolerability and activity of combined LEN/AZA administration in post-transplantation relapse.

**PATIENTS AND METHODS:**

Twenty-nine patients who had relapsed after allo-SCT for AML (n = 24) or MDS (n = 5) were treated with sequential AZA (75 mg/m^2^ for 7 days) followed by escalating doses of LEN on days 10 to 30. Dose allocation and maximum tolerated dose (MTD) estimation were guided by a modified Bayesian continuous reassessment method (CRM).

**RESULTS:**

Sequential AZA and LEN therapy was well tolerated. The MTD of post-transplantation LEN, in combination with AZA, was determined as 25 mg daily. Three patients developed grade 2 to 4 GVHD. There was no GVHD-related mortality. Seven of 15 (47%) patients achieved a major clinical response after LEN/AZA therapy. CD8+ T cells demonstrated impaired interferon-γ/tumor necrosis factor–α production at relapse, which was not reversed during LEN/AZA administration.

**CONCLUSION:**

We conclude LEN can be administered safely post-allograft in conjunction with AZA, and this combination demonstrates clinical activity in relapsed AML/MDS without reversing biologic features of T-cell exhaustion. The use of a CRM model delivered improved efficiency in MTD assessment and provided additional flexibility. Combined LEN/AZA therapy represents a novel and active salvage therapy in patients who had relapsed post-allograft.

## INTRODUCTION

Allogeneic stem-cell transplantation (allo-SCT) plays an increasingly important role in the management of acute myeloid leukemia (AML) and myelodysplasia (MDS) consequent on the advent of reduced-intensity conditioning regimens and increased availability of alternative donors.^1^ However, 30% to 80% of patients receiving allografts for AML are destined to relapse, and fewer than 10% survive long term.^2^ Consequently, relapse is now the major cause of treatment failure in patients receiving allografts for AML/MDS.^3^ Although a second allograft and donor lymphocyte infusion (DLI) both have the capacity to deliver durable survival in patients with recurrent disease, they are only effective in patients who achieve a morphologic complete remission (CR) after salvage therapy.^4,5^ Currently, salvage options are highly unsatisfactory. Intensive chemotherapy results in acquisition of a CR in a proportion of patients who had relapsed post-transplantation, but it is toxic and often poorly tolerated.^6^ As a consequence, most patients who relapse after an allograft are palliated, and the development of effective salvage regimens represents a major unmet need.

Recently, both the DNA methyltransferase inhibitor azacitidine (AZA) and the immunomodulatory drug lenalidomide (LEN) have been shown to possess significant antileukemic activity in newly diagnosed AML and benefit from a broadly favorable toxicity profile.^7,8^ In patients who relapse after allo-SCT, AZA is well tolerated, and 15% to 20% of patients achieve a CR after a median of 108 days from the commencement of AZA therapy.^9^ Strategies with the ability to increase the activity of AZA monotherapy are therefore required. Although low-dose LEN demonstrates antileukemic activity in patients who relapse after allo-SCT, it is associated with high rates of severe, often life-threatening, graft-versus-host disease (GVHD), and its administration is generally viewed to be contraindicated post-transplantation.^10,11^ In addition to its antileukemic activity, AZA accelerates reconstitution of T-regulatory cells post-transplantation in murine models, resulting in a reduced risk of severe GVHD.^12^ These observations have been replicated in patients allografted for AML.^13-15^ We therefore hypothesized that coadministration of AZA may deliver additive antileukemic activity while serving to ameliorate the risk of severe GVHD associated with LEN administration post-transplantation.

A major factor limiting the expeditious examination of novel drug combinations in complex clinical settings, such as post-transplantation relapse, has been the limitations of standard early-phase trial designs conventionally used to establish the maximum tolerated dose (MTD).^16,17^ Emerging data have highlighted the superior performance of model-based designs, such as the continuous reassessment method (CRM), in correctly identifying the MTD by permitting more efficient patient allocation, thereby enabling a more rapid progression to later phases of clinical trial assessment.^18,19^ We therefore examined the tolerability and activity of combined LEN/AZA therapy in patients who had relapsed after allo-SCT for AML using dose transition pathways (DTP). DTP is a useful design calibration tool used to provide a tailored CRM design integrating important clinical judgements in a revised statistical model, ensuring applicability in practice before implementation, and an operational tool to guide the process of making dose escalation/de-escalation decisions.^20^

## PATIENTS AND METHODS

### Eligibility

The VIOLA trial (ISCRCTN98163167, EudraCT 2013-002118-11) was delivered by the Bloodwise Trials Acceleration Program as a prospective, open-label, phase I dose-finding, multicenter trial designed to determine the MTD of LEN in combination with AZA in patients who have relapsed after allo-SCT for AML or MDS. Patients with active acute or chronic GVHD or a history of grade 3 or 4 GVHD were excluded. Impaired renal or hepatic function (defined as total bilirubin greater than or equal to 2.5 times the upper limit of normal (ULN), aspartate aminotransferase or alanine aminotransferase greater than or equal to 3.0 times the ULN, and estimated glomerular filtration rate less than or equal to 40 mL/min) were exclusion criteria. Patients receiving immunosuppressive therapies at the time of relapse and those who had received antitumor therapies within 28 days before the start of protocol treatment were also excluded.

### Trial Design and Assessment of Safety and Response

Patients were recruited sequentially in planned cohort sizes of three, with a maximum sample size of 30. AZA (75 mg/m^2^) was administered by subcutaneous injection on a 5 + 2 + 2 schedule, commencing on day 1 of a 42-day cycle for up to six cycles. A predetermined dose level of LEN was administered from days 10 to 30, followed by a 12-day rest period according to a modified one-stage, one-parameter Bayesian CRM (Table 1). Tolerability and safety were assessed according to the National Cancer Institute Common Terminology Criteria for Adverse Events v4.0 for all patients who commenced treatment. Response to trial therapy was assessed using modified Cheson criteria.^21^ Patients achieving a major clinical response—defined as a CR, CR with incomplete blood count recovery, or partial response (PR)—within the first six cycles of treatment were permitted to continue study treatment until loss of response. Patients who were nonresponding discontinued trial therapy. A dose-limiting toxicity (DLT) was defined as the occurrence of grade 3 or 4 acute GVHD, recurrent grade 2 acute GVHD, any increase in GVHD grade within the first two cycles of therapy, or grade 2 GVHD persisting for 42 or more days. New-onset grade 3 or 4 nonhematologic toxicity or death related to treatment were also considered to be DLTs and were formally evaluated up to the end of cycle 1 treatment (day 30).

**TABLE 1.**
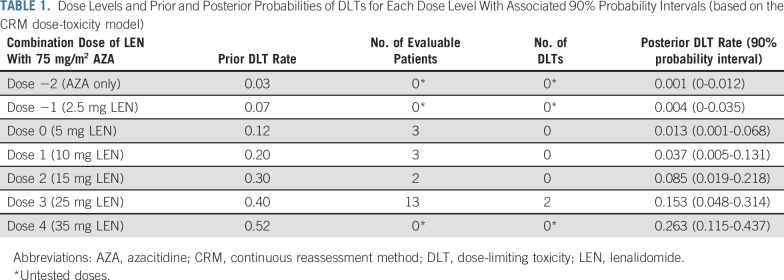
Dose Levels and Prior and Posterior Probabilities of DLTs for Each Dose Level With Associated 90% Probability Intervals (based on the CRM dose-toxicity model)

### CRM Model for Assessment of the MTD

An empirical dose-toxicity model was used to calculate estimates of the probability of occurrence of DLT for the investigated doses and recommended dose escalation/de-escalation on the basis of the investigators’ experience and published data (Table 1).^18,22^ A normal prior of mean 0 and variance 0.75 for the slope parameter was assumed. The prior guess of MTD was dose 1, but given the reports of severe GVHD occurring with relatively low doses of LEN post-transplantation, a cautious starting dose for the first cohort of patients was decided at dose 0. On the basis of the DLT outcomes of the first cohort, posterior DLT probabilities at all dose levels were computed. The recommended dose level for the second cohort was the dose with updated DLT rate closest to the target level of 20%, incorporating any additional restrictions, such as no skipping of doses in escalation. This continued for the subsequent cohorts until the maximum sample size was reached or if stopped early. Given the complexity of the trial population under study, the proposed CRM design incorporated practical modifications to create a tailored model, allowing the trial to stop early if there are at least 12 patients who recieved a dosage at the MTD level and there is strong evidence that the lowest dose is too toxic. Using the DTP tool, the Bayesian stopping-early criterion was calibrated such that the trial stopped early if there was a high chance (greater than 72%) that the posterior probability of DLT at the lowest dose was more than 10% greater than the target DLT rate of 20%.^20^ For CRM modeling, source code obtained from R package dfcrm was modified to incorporate practical modifications to the design.

### Immune Evaluation

The frequency of CD3+ T cells and CD4+FOXP3+ T-regulatory cells was analyzed in all samples by a CyAn flow Cytometer (Beckman Coulter, High Wycombe, Buckinghamshire, UK). Expression of CD3, CD4, and forkhead box 3 (FOXP3) (BioLegend, San Diego, California, USA) in combination with Programmed cell death protein 1 (PD1), Lymphocyte-activation gene 3 (LAG3), and T-cell immunoglobulin and mucin-domain containing-3 (BioLegend) exhaustion markers were investigated on frozen peripheral blood mononuclear cell samples. To assess the activation status of these T cells, 0.5 × 10^6^ peripheral-blood mononuclear cells were treated with 20 ng/mL phorbol 12-myristate 13-acetate (Sigma, St Louis, Missouri, USA) and 500 ng/mL ionomycin (Sigma) in vitro for 48 hours. Supernatants were harvested and the cytokine profile was analyzed by bead-array flow cytometry (BioLegend).

### Statistical Estimation

The MTD was defined as the dose level with an estimated DLT rate closest to 20% (target) with its associated DLT rate and 90% probability interval. The DLT evaluable population included those patients who had received all 7 days of AZA and at least 17 of 21 days of LEN in cycle 1. The overall survival (OS) curves for responders and nonresponders were generated using Kaplan-Meier plot and compared using the log-rank test. For immune evaluation analysis, two-tailed Mann-Whitney *U* test or Wilcoxon signed-rank test for unpaired and paired samples, respectively, were performed. Statistical analyses were conducted using R version 3.4.2 and GraphPad Prism version 6.0h (GraphPad Software, La Jolla CA). *P* values < .05 were considered statistically significant.

## RESULTS

### Patient Characteristics

**FIG 1.**
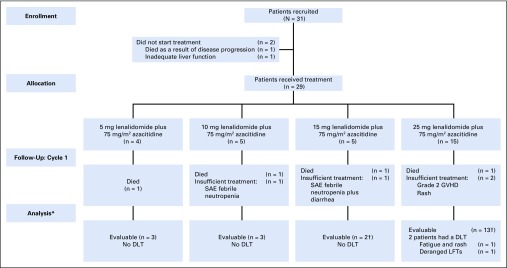
Flow diagram of participants summarizing patient flow from trial entry, detailing evaluability for maximum tolerated dose (MTD) determination. (*) A patient was considered to be evaluable for MTD determination if they received all seven doses of azacitidine and at least 80% (17 days) of lenalidomide in cycle 1 of treatment; those who withdrew/died for reasons that were deemed to be non–treatment related in cycle 1 were classed as inevaluable and were replaced accordingly. (†) One patient was treated incorrectly with the higher dose (25 mg lenalidomide plus 75 mg/m^2^ azacitidine) for cycle 1 rather than 15 mg lenalidomide plus 75 mg/m^2^ azacitidine and so was included in the higher dose level for the analysis. The patient reverted to the correct assigned dose level for the proceeding cycles. DLT, dose-limiting toxicity; GVHD, graft-versus-host disease; LFT, liver function tests; SAE, serious adverse event.

Thirty-one patients who had relapsed after an allograft for AML or MDS were recruited between February 2014 and December 2016. Twenty-nine patients commenced trial therapy (AML, n = 24; MDS, n = 5; Fig 1; Table 2) with a median follow-up of 23 months. The mean percentage blasts at commencement of treatment was 40%, and the median time from transplantation to relapse before the trial was 10 months. Patients received a median of three cycles of therapy in the study (range, 0 to 11).

**TABLE 2.**
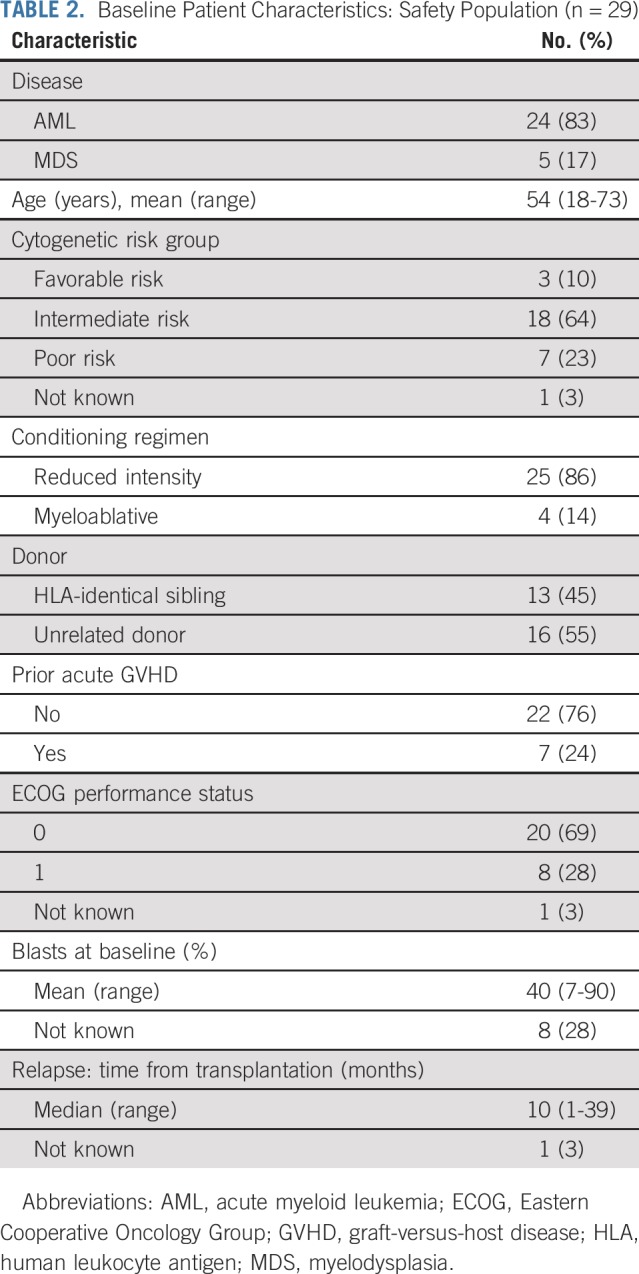
Baseline Patient Characteristics: Safety Population (n = 29)

### MTD Assessment

**FIG A1.**
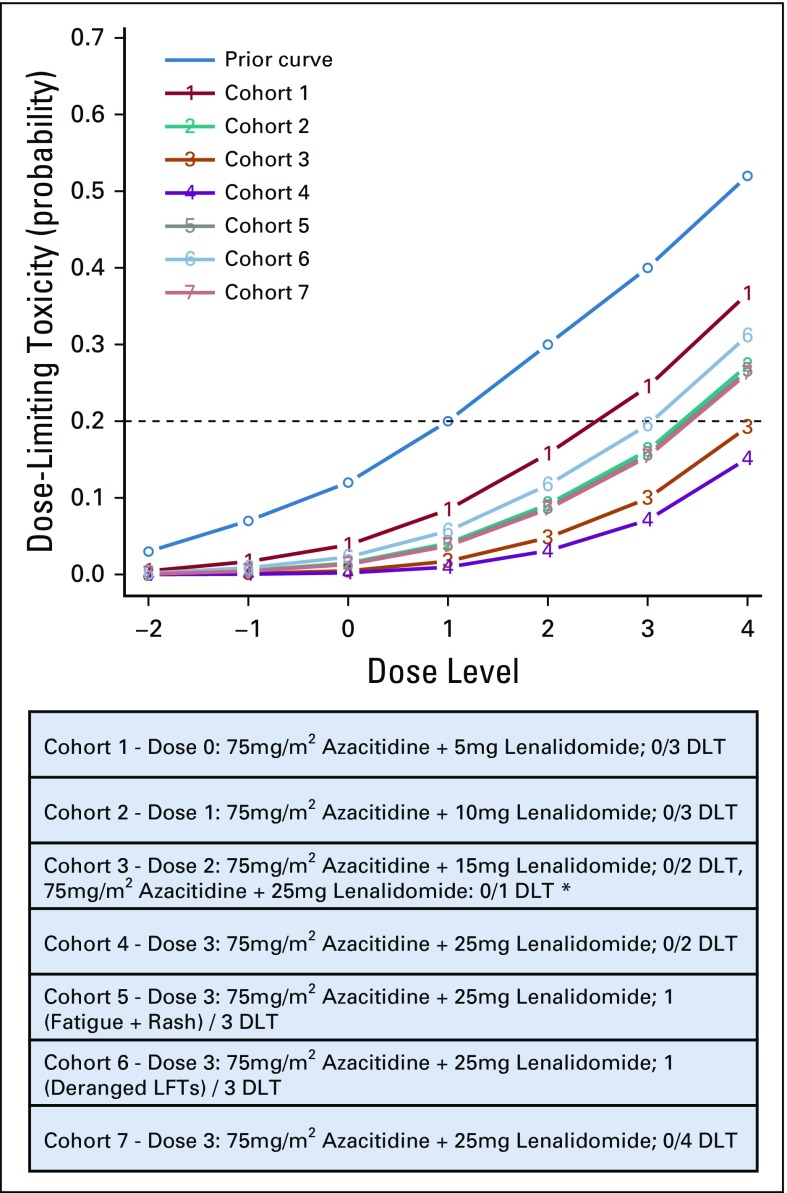
Continual reassessment model dose-toxicity curves. This figure shows how the estimates of the probabilities of dose-limiting toxicities (DLTs) at each dose evolve from the initial guesses of DLT (prior curve) to the final cohort 7. The dose-toxicity curves initially dropped from cohorts 1 to 4 (because no DLT was observed at all tested doses) and only increased when DLTs were observed for cohorts 5 and 6 at 25 mg lenalidomide. (*) One patient was incorrectly treated with dose level 3 in cycle 1.

**FIG A2.**
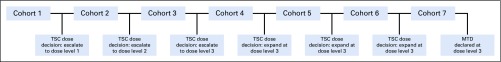
Trial Steering Committee (TSC) dose decisions by cohort. MTD, maximum tolerated dose.

Twenty-one patients were evaluable for MTD assessment (Fig 1). Sequential updates of the estimated dose-toxicity curves were determined using the CRM (from initial prior curve) after each cohort (Appendix Fig A1, online only), and the final model’s estimated posterior probabilities of DLT for each dose level and their associated 90% probability intervals were calculated (Table 1). The MTD for LEN when administered with AZA was determined as 25 mg on the basis of an estimated posterior probability of DLT of 15.3% (90% probability interval, 4.8% to 31.4%). Thirteen of 21 (62%) evaluable patients were treated at the MTD. The maximum likelihood estimate of the DLT rate at the model-determined MTD and an associated 95% CI (Clopper-Pearson) is 15.4% (1.9% to 45.5%). Sequential dose decisions made by the Trial Steering Committee after each cohort are presented in Appendix Figure A2 (online only).

### Assessment of Safety

Twenty treatment-related nonhematologic toxicities occurred in more than 10% of patients at grade 3 or greater at any time from cycle 1 through all cycles of treatment (Table 3). Three patients developed acute GVHD during therapy. One developed grade 2 lower gut GVHD after receiving four cycles of LEN at a dose of 15 mg. Two patients receiving 25 mg LEN developed GVHD: one with grade 2 acute skin GVHD in cycle 1 and one with grade 3 liver GVHD during cycle 6 of therapy. All patients who developed GVHD responded to steroid therapy, and there were no GVHD-related deaths. Two DLTs occurred in patients receiving 25 mg of LEN. One patient developed fatigue and rash (grade 3); the other experienced deranged liver function tests (grade 3). Twenty-two patients died: 19 from resistant or progressive disease and three from infectious complications.

**TABLE 3.**
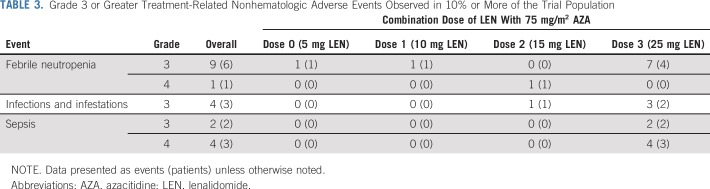
Grade 3 or Greater Treatment-Related Nonhematologic Adverse Events Observed in 10% or More of the Trial Population

### Clinical Response and OS

**FIG A3.**
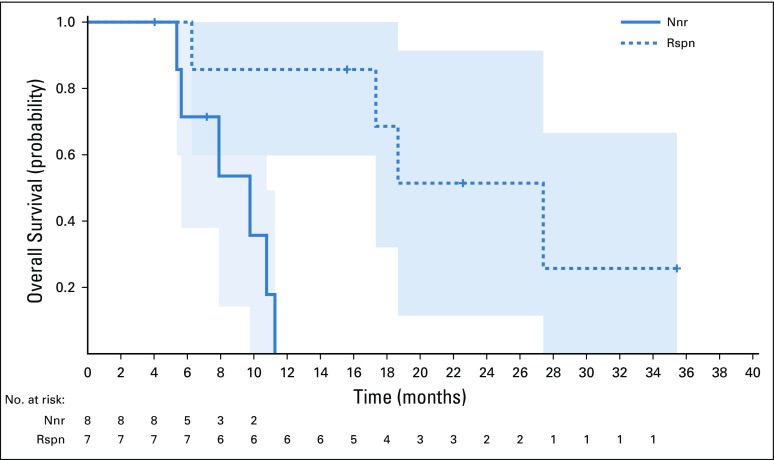
Overall survival: responders/nonresponders. Kaplan-Meier overall survival curves for non-responders (solid line) and responders (complete remission/complete remission with incomplete blood count recovery/partial response; dashed line) were assessed in 15 evaluable patients who received at least three cycles of treatment. Overall survival time was seen to be greater in those patients who responded to treatment (*P* = .004). Nnr, non-responders; Rspn, responders.

Seven of 15 (47%) patients who received at least three cycles of treatment achieved a major clinical response to LEN/AZA salvage (CR, n = 3; CR with incomplete blood count recovery, n = 3; PR, n = 1). Of the seven responders, six patients had AML and one MDS. The mean age was 52 years (range, 28 to 68 years). The mean percent blasts at baseline was 18% (range, 7% to 34%). The median time from transplantation to relapse was 11 months (range, 4 to 31 months). There was no correlation between the administered dose of LEN and the response rate (Table 4). Five patients who achieved a major clinical response subsequently received DLI (n = 3) or a second allograft (n = 2). Of the three patients who received DLI, two patients are still alive 15 and 35 months after trial therapy commenced. One patient is still alive after a second transplantation, 22 months after commencing trial therapy. The median OS in patients who responded to treatment was 27 months, compared with 10 months in nonresponders (*P* = .004; Appendix Fig A3, online only). In the total cohort of 29 patients receiving at least one dose of LEN/AZA, the response rate was 24% (seven of 29), which likely reflected limited exposure to trial therapy, most commonly because of progressive disease.

**TABLE 4.**
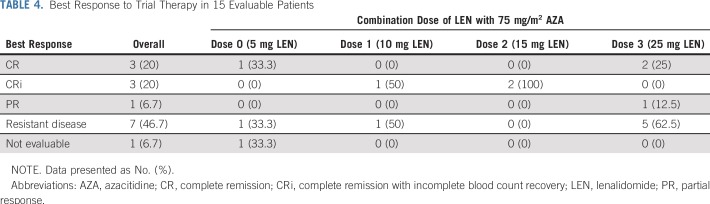
Best Response to Trial Therapy in 15 Evaluable Patients

### Immune Evaluation

**FIG 2.**
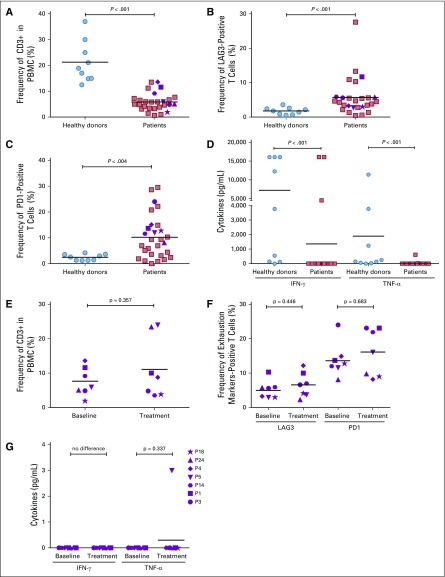
Cell numbers and cytokine expression in healthy controls and trial patients. (A) The frequency of CD3+ T cells in peripheral-blood mononuclear cells (PBMCs) from healthy donors and trial patients. (B) Expression of T-cell surface marker Lymphocyte-activation gene 3 (LAG3) in healthy donors and trial patients. (C) Expression of T-cell surface marker Programmed cell death protein 1 (PD1) in healthy donors and trial patients. (D) Expression of T helper 1 cells (Th1) cytokines interferon (IFN)-γ and tumor necrosis factor (TNF)-α after phorbol 12-myristate 13-acetate ionomycin stimulation in healthy donors and trial patients. (E) Frequency of CD3+ T cells before and after commencement of trial therapy in patients demonstrating a clinical response to trial therapy. (F) Functional status of T cells in patients who demonstrated a clinical response to trial therapy as determined by LAG3 and PD1 expression. (G) Expression of Th1 cytokines IFN-γ and TNF-α after phorbol 12-myristate 13-acetate ionomycin stimulation in patients achieving a clinical response to trial therapy.

Exposure to both AZA and LEN can induce differences in T-cell activation and function in vitro, and we therefore sequentially characterized the number and phenotype of CD3+ T cells in patients before and after trial therapy. A significant reduction in the frequency of T cells was observed before commencement of trial therapy, compared with healthy controls (*P* < .001; Fig 2A). T cells from trial patients at baseline expressed increased LAG3 and PD1, consistent with an exhaustion phenotype (Figs 2B and 2C). No CD4+FOXP3+ T cells were identified. T cells from trial patients demonstrated a significant reduction in release of T helper 1 cells cytokines interferon-γ and tumor necrosis factor–α after phorbol 12-myristate 13-acetate-ionomycin stimulation (*P* < .001) compared with healthy controls (Fig 2D). Release of proinflammatory cytokines IL-2, IL-5, and IL-6 were similarly reduced (Appendix Figs 4A-4C, online only).

The seven patients who demonstrated a clinical response (CR or PR) to combined AZA and LEN trial therapy were additionally evaluated. The CD3+ T-cell frequency in the peripheral blood did not increase after six cycles of trial therapy (Fig 2E). No significant changes in the frequency of PD1/LAG3+ T cells (Fig 2F) or cytokine profile (Fig 2G) were identified after trial therapy. Taken together, these findings indicate that T-cell function is impaired in patients who relapse post-allograft consistent with an exhaustion phenotype, and that LEN/AZA treatment exerts antitumor activity independent of this pathway.

## DISCUSSION

The demonstration that high doses of LEN, in combination with AZA, exert significant antileukemic activity with modest toxicity in patients who relapse after allo-SCT identifies a potentially important new salvage strategy in patients who relapse post-transplantation. Intensive chemotherapy is often ineffective in this setting and associated with substantial toxicity. Although the targeted therapies sorafenib and AG221 demonstrate significant clinical activity in patients with relapsed disease post-allograft their use is restricted to specific biologic subtypes of AML.^23,24^ Our data, for the first time to our knowledge, demonstrate that LEN can be safely administered post-allograft in combination with AZA, and seems to be associated with a higher CR/PR rate than AZA monotherapy.^9^ The observed clinical activity may be consequent on the additive antileukemic activity of LEN and AZA or alternatively represent pharmacologic manipulation of the graft-versus-leukemia effect. AZA has previously been shown to up-regulate tumor antigen expression on AML blasts and can also induce a CD8+ T-cell response post-allograft, whereas LEN has direct T-cell activation properties.^13^

A striking observation in this study was the tolerability of LEN after allo-SCT. Previous studies using LEN as maintenance or pre-emptive therapy in the first 3 months post-transplantation reported excessive rates of severe GVHD after LEN administration, even at modest doses of 5 to 10 mg. In contrast, in this study patients tolerated doses of up to 25 mg LEN, in conjunction with AZA, with acceptable rates of GVHD. Why then might this study have yielded results different from previous reports? It may be relevant that the majority of patients treated with LEN maintenance in previous studies were recipients of T-replete allografts, whereas patients in this series received either alemtuzumab or anti-thymocyte globulin as GVHD prophylaxis. Alternatively, the coadministration of AZA may augment T-regulatory cell expansion, which may decrease the risk of GVHD, as observed in mouse models.^12^ It will therefore be of interest to explore whether AZA coadministration reduces the risk of GVHD when LEN is administered early post-transplantation.

**FIG A5.**
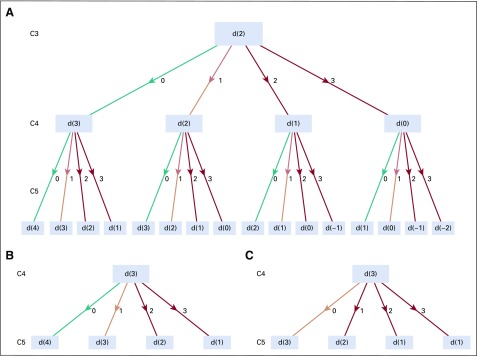
Dose transition pathways (DTP) flow diagrams. Flow diagrams presenting the different pathways for dose (d) recommendations on the basis of the possible patient outcome permutations within the trial for future cohorts (C). (A) DTP for C3 to C5 on the basis of no observed DLTs for C1 and C2: (zero of three at dose 0 and zero of three at dose 1). The recommended dose for C3 is dose 2. The DTP for subsequent cohorts only apply if the cohort size is three at the recommended dose. (B, C) Two scenarios where this is not the case. (B) Scenario in which C3 consist of two patients who received dose level 2 and one patient received dose level 3, with no dose-limiting toxicities (DLTs) experienced. (C) Scenario in which C3 consist of two patients who received dose level 2 and one patient received dose level 3, with no DLT at dose level 2 but a DLT experienced by the patient who received dose level 3.

To obtain the MTD in such a complex patient population, we adopted a tailored CRM design that integrates important clinical judgements. The CRM provides greater accuracy in its determination of the MTD compared with common rule-based designs and both Modified Toxicity Probability Interval and Bayesian Optimal Interval.^19,25^ Implementing an early stopping criterion that permitted clinical judgement in this population was also of value to manage excessive DLTs seen at the starting and lower doses in addition to having favorable statistical properties. This is enabled via use of a novel investigator-oriented tool, the DTP, which maps out dose decisions in advance. This is the first trial, to our knowledge, that has implemented a tailored CRM coupled with DTP, translating a complex dose-finding design to simple decision making for trialists. Benefits of using this innovative design were seen not only at the design stage but also during the running of the trial with the ease of visualization of dose pathways simplifying the statistical black box of complex designs. In addition, assessment of the MTD in this high-risk patient population was accelerated by the adoption of such a flexible design, which coped effectively with the challenges of unexpected dosing error and cohort size variation due to early patient dropout, not necessarily requiring replacement of all patients who were inevaluable. Because such unintended deviations could lead to different pathways for subsequent cohorts (which were originally planned for cohort size of three at the model’s recommended doses), the DTP could be easily updated (Appendix Fig A5 [online only] provides a flow diagram of a DTP and two possible updates). These have collectively led to substantial savings in time and resources. Such unforeseen occurrences would have been difficult for a conventional 3 + 3 design. At the point of the final determination of the MTD, 62% (13 of 21) patients had been treated at the MTD. Implementation of the CRM allowed for more accurate determination of the MTD while treating the majority of patients at an optimal dose level.

T cells are a major arm of the anticancer immune response post-allograft, yet controversy remains over their functional state in AML.^26,27^ In a smaller cohort, T-cell exhaustion in patients with AML may predict relapse post–allo-SCT.^28^ Correspondingly, we have shown that the T cells in patients post-transplantation have an exhaustion phenotype manifested by increased PD1 and LAG3 expression, in combination with reduced interferon-γ, tumor necrosis factor–α, IL-2, IL-5, and IL-6 secretion. We show that combined AZA/LEN therapy does not reverse the T-cell phenotype, and T-cell status does not correlate with response to these agents post-allograft. The findings extend our previous results that AML creates an immunosuppressive microenvironment to T cells.^29^ Alternative approaches to reverse T-cell exhaustion could be an adjunct to enhance post-transplantation immune surveillance for patients with AML.

Our data establish a potentially important role for an LEN/AZA combination as salvage therapy in patients with relapsed AML post-allograft and support a randomized comparison of this novel regimen with intensive chemotherapy in this area of major unmet need. Alternatively, combined LEN/AZA therapy could be administered either as maintenance therapy^15^ or, because of the requirement for at least three cycles of therapy to maximize response, pre-emptively in patients with evidence of measurable residual disease.

## Figures and Tables

**FIG A4.**
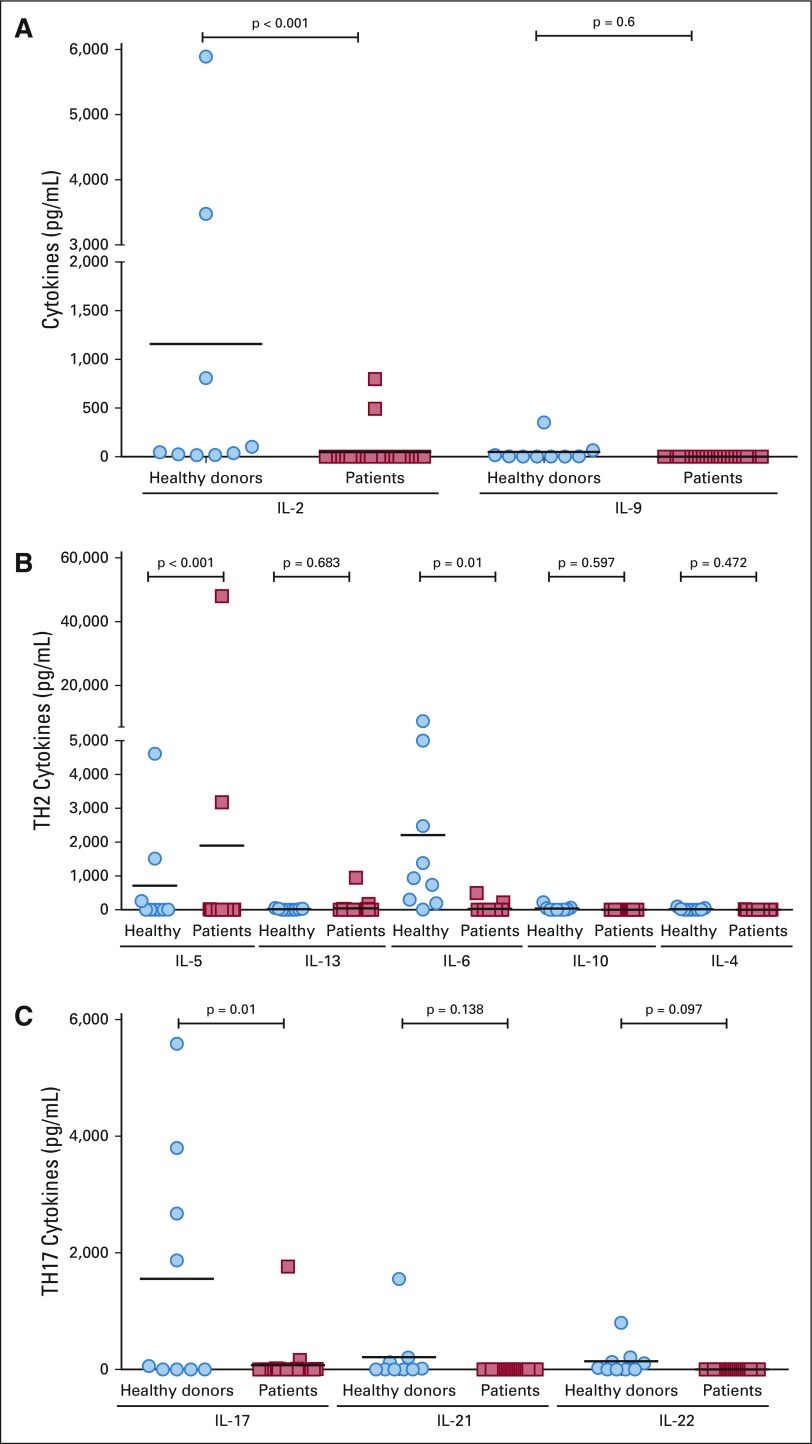
Expression of proinflammatory cytokines in healthy donors and patients with acute myeloid leukemia (AML). Release of proinflammatory cytokines IL-2, IL-5, and IL-6 were reduced in the patients with AML compared with healthy donors. (A) Concentrations of IL-2 and Il-9 in the supernatants of stimulated peripheral blood mononuclear cells (PBMCs) from all patients. (B) Concentrations of T helper 2 cells (Th2) cytokines in the supernatants of stimulated PBMCs from all patients. (C) Concentrations of Th17 cytokines in the supernatants of stimulated PBMCs from all patients.

## References

[1] Cornelissen JJ Gratwohl A Schlenk RF et al: The European LeukemiaNet AML Working Party consensus statement on allogeneic HSCT for patients with AML in remission: An integrated-risk adapted approach. Nat Rev Clin Oncol 9:579-590, 2012 22949046 10.1038/nrclinonc.2012.150

[2] Craddock C Versluis J Labopin M et al: Distinct factors determine the kinetics of disease relapse in adults transplanted for acute myeloid leukaemia. J Intern Med 283:371-379, 2018 29214689 10.1111/joim.12720

[3] Schmid C Labopin M Nagler A et al: Treatment, risk factors, and outcome of adults with relapsed AML after reduced intensity conditioning for allogeneic stem cell transplantation. Blood 119:1599-1606, 2012 22167752 10.1182/blood-2011-08-375840

[4] Schmid C Labopin M Nagler A et al: Donor lymphocyte infusion in the treatment of first hematological relapse after allogeneic stem-cell transplantation in adults with acute myeloid leukemia: A retrospective risk factors analysis and comparison with other strategies by the EBMT Acute Leukemia Working Party. J Clin Oncol 25:4938-4945, 2007 17909197 10.1200/JCO.2007.11.6053

[5] Christopeit M Kuss O Finke J et al: Second allograft for hematologic relapse of acute leukemia after first allogeneic stem-cell transplantation from related and unrelated donors: The role of donor change. J Clin Oncol 31:3259-3271, 2013 23918951 10.1200/JCO.2012.44.7961

[6] Motabi IH Ghobadi A Liu J et al: Chemotherapy versus hypomethylating agents for the treatment of relapsed acute myeloid leukemia and myelodysplastic syndrome after allogeneic stem cell transplant. Biol Blood Marrow Transplant 22:1324-1329, 2016 27026249 10.1016/j.bbmt.2016.03.023

[7] Dombret H Seymour JF Butrym A et al: International phase 3 study of azacitidine vs conventional care regimens in older patients with newly diagnosed AML with >30% blasts. Blood 126:291-299, 2015 25987659 10.1182/blood-2015-01-621664 PMC4504945

[8] Fehniger TA Uy GL Trinkaus K et al: A phase 2 study of high-dose lenalidomide as initial therapy for older patients with acute myeloid leukemia. Blood 117:1828-1833, 2011 21051557 10.1182/blood-2010-07-297143 PMC3318598

[9] Craddock C Labopin M Robin M et al: Clinical activity of azacitidine in patients who relapse after allogeneic stem cell transplantation for acute myeloid leukemia. Haematologica 101:879-883, 2016 27081178 10.3324/haematol.2015.140996 PMC5004468

[10] Sockel K Bornhaeuser M Mischak-Weissinger E et al: Lenalidomide maintenance after allogeneic HSCT seems to trigger acute graft-versus-host disease in patients with high-risk myelodysplastic syndromes or acute myeloid leukemia and del(5q): Results of the LENAMAINT trial. Haematologica 97:e34-e35, 2012 22952334 10.3324/haematol.2012.067629 PMC3436225

[11] Kneppers E van der Holt B Kersten MJ et al: Lenalidomide maintenance after nonmyeloablative allogeneic stem cell transplantation in multiple myeloma is not feasible: Results of the HOVON 76 Trial. Blood 118:2413-2419, 2011 21690556 10.1182/blood-2011-04-348292

[12] Choi J Ritchey J Prior JL et al: In vivo administration of hypomethylating agents mitigate graft-versus-host disease without sacrificing graft-versus-leukemia. Blood 116:129-139, 2010 20424188 10.1182/blood-2009-12-257253 PMC2904576

[13] Goodyear OC Dennis M Jilani NY et al: Azacitidine augments expansion of regulatory T cells after allogeneic stem cell transplantation in patients with acute myeloid leukemia (AML). Blood 119:3361-3369, 2012 22234690 10.1182/blood-2011-09-377044

[14] Schroeder T Fröbel J Cadeddu RP et al: Salvage therapy with azacitidine increases regulatory T cells in peripheral blood of patients with AML or MDS and early relapse after allogeneic blood stem cell transplantation. Leukemia 27:1910-1913, 2013 23519388 10.1038/leu.2013.64

[15] Craddock C Jilani N Siddique S et al: Tolerability and clinical activity of post-transplantation azacitidine in patients allografted for acute myeloid leukemia treated on the RICAZA trial. Biol Blood Marrow Transplant 22:385-390, 2016 26363443 10.1016/j.bbmt.2015.09.004 PMC4728172

[16] Rogatko A Schoeneck D Jonas W et al: Translation of innovative designs into phase I trials. J Clin Oncol 25:4982-4986, 2007 17971597 10.1200/JCO.2007.12.1012

[17] Nie L Rubin EH Mehrotra N et al: Rendering the 3 + 3 design to rest: More efficient approaches to oncology dose-finding trials in the era of targeted therapy. Clin Cancer Res 22:2623-2629, 2016 27250933 10.1158/1078-0432.CCR-15-2644

[18] O’Quigley J Pepe M Fisher L: Continual reassessment method: a practical design for phase 1 clinical trials in cancer. Biometrics 46:33-48, 1990 2350571

[19] Iasonos A Wilton AS Riedel ER et al: A comprehensive comparison of the continual reassessment method to the standard 3 + 3 dose escalation scheme in Phase I dose-finding studies. Clin Trials 5:465-477, 2008 18827039 10.1177/1740774508096474 PMC2637378

[20] Yap C Billingham LJ Cheung YK et al: Dose transition pathways: The missing link between complex dose-finding designs and simple decision-making. Clin Cancer Res 23:7440-7447, 2017 28733440 10.1158/1078-0432.CCR-17-0582

[21] Cheson BD Bennett JM Kopecky KJ et al: Revised recommendations of the International Working Group for Diagnosis, Standardization of Response Criteria, Treatment Outcomes, and Reporting Standards for Therapeutic Trials in Acute Myeloid Leukemia. J Clin Oncol 21:4642-4649, 2003 14673054 10.1200/JCO.2003.04.036

[22] Goodman SN Zahurak ML Piantadosi S: Some practical improvements in the continual reassessment method for phase I studies. Stat Med 14:1149-1161, 1995 7667557 10.1002/sim.4780141102

[23] Stein EM DiNardo CD Pollyea DA et al: Enasidenib in mutant *IDH2* relapsed or refractory acute myeloid leukemia. Blood 130:722-731, 2017 28588020 10.1182/blood-2017-04-779405 PMC5572791

[24] Sharma M Ravandi F Bayraktar UD et al: Treatment of FLT3-ITD-positive acute myeloid leukemia relapsing after allogeneic stem cell transplantation with sorafenib. Biol Blood Marrow Transplant 17:1874-1877, 2011 21767516 10.1016/j.bbmt.2011.07.011 PMC4061979

[25] Horton BJ Wages NA Conaway MR: Performance of toxicity probability interval based designs in contrast to the continual reassessment method. Stat Med 36:291-300, 2017 27435150 10.1002/sim.7043 PMC5267938

[26] Kong Y Zhu L Schell TD et al: T-cell immunoglobulin and ITIM domain (TIGIT) associates with CD8+ T-cell exhaustion and poor clinical outcome in AML patients. Clin Cancer Res 22:3057-3066, 2016 26763253 10.1158/1078-0432.CCR-15-2626

[27] Schnorfeil FM Lichtenegger FS Emmerig K et al: T cells are functionally not impaired in AML: Increased PD-1 expression is only seen at time of relapse and correlates with a shift towards the memory T cell compartment. J Hematol Oncol 8:93, 2015 26219463 10.1186/s13045-015-0189-2 PMC4518596

[28] Kong Y Zhang J Claxton DF et al: PD-1(hi)TIM-3(+) T cells associate with and predict leukemia relapse in AML patients post allogeneic stem cell transplantation. Blood Cancer J 5:e330, 2015 26230954 10.1038/bcj.2015.58 PMC4526784

[29] Mussai F De Santo C Abu-Dayyeh I et al: Acute myeloid leukemia creates an arginase-dependent immunosuppressive microenvironment. Blood 122:749-758, 2013 23733335 10.1182/blood-2013-01-480129 PMC3731930

